# Short‐term pre‐operative high‐intensity interval training does not improve fitness of colorectal cancer patients

**DOI:** 10.1111/sms.13460

**Published:** 2019-05-29

**Authors:** Catherine L. Boereboom, James E. M. Blackwell, John P. Williams, Bethan E. Phillips, Jonathan N. Lund

**Affiliations:** ^1^ MRC‐ARUK Centre for Musculoskeletal Ageing Research, Royal Derby Hospital Centre University of Nottingham Derby UK; ^2^ National Institute for Health Research Nottingham Biomedical Research Centre Queens Medical Centre Nottingham UK; ^3^ Surgical Department Royal Derby Hospital Derby UK; ^4^ Anaesthetic Department Royal Derby Hospital Derby UK

**Keywords:** colorectal neoplasms, exercise, pre‐operative therapy, surgery

## Abstract

**Background:**

Pre‐operative cardiorespiratory fitness (CRF) in colorectal cancer (CRC) patients has been shown to affect post‐operative outcomes. The aim of this study was to test the feasibility of high‐intensity interval training (HIIT) for improving fitness in pre‐operative CRC patients within the 31‐day cancer waiting‐time targets imposed in the UK.

**Methods:**

Eighteen CRC patients (13 males, mean age: 67 years (range: 52‐77 years) participated in supervised HIIT on cycle ergometers 3 or 4 times each week prior to surgery. Exercise intensity during 5 × 1‐minute HIIT intervals (interspersed with 90‐second recovery) was 100%‐120% maximum wattage achieved at a baseline cardiopulmonary exercise test (CPET). CPET before and after HIIT was used to assess CRF.

**Results:**

Patients completed a mean of eight HIIT sessions (range 6‐14) over 19 days (SD 7). There was no significant increase in VO_2_ peak (23.9 ± 7.0 vs 24.2 ± 7.8 mL/kg/min (mean ± SD), *P* = 0.58) or anaerobic threshold (AT: 14.0 ± 3.4 vs 14.5 ± 4.5 mL/kg/min, *P* = 0.50) after HIIT. There was a significant reduction in resting systolic blood pressure (152 ± 19 vs 142 ± 19 mm Hg, *P* = 0.0005) and heart rate at submaximal exercise intensities after HIIT.

**Conclusions:**

Our pragmatic HIIT exercise protocol did not improve the pre‐operative fitness of CRC patients within the 31‐day window available in the UK to meet cancer surgical waiting‐time targets.

## 1 INTRODUCTION

There is a growing body of evidence that objective measures of pre‐operative cardiorespiratory fitness (CRF) correlate with post‐operative outcomes following elective colorectal cancer (CRC) surgery^1^, ^2^, ^3^ and that threshold variables exist for cardiopulmonary exercise testing (CPET, the “gold‐standard” text of cardiorespiratory fitness^4^) below which complications are more likely to occur.^5^ These thresholds exist for two of the most commonly derived CPET parameters, namely peak oxygen consumption (VO_2_ peak: the peak value for oxygen consumption by an individual during a period of exercise) and anaerobic threshold (AT: the point at which, during increasing work, the aerobic metabolism of an individual is supplemented by the anaerobic metabolism to produce cellular energy^6^). Although exercise training is well known to improve CRF in a variety of healthy and clinical cohorts,^7^ and therefore has the potential to move people away from these “at‐risk” thresholds, it is not yet known whether this can be achieved by CRC patients within the maximum 31‐day target time frame available in the UK between decision to treat and operation.^8^

Several studies have shown pre‐operative improvements in other aspects of physical function (typically walking capacity)^2^, ^9^ with exercise training, but reports of changes in CPET parameters are rare.^10^ This study differs from previous work in this area by using objective CPET measures before and after high‐intensity interval training (HIIT) to explore the feasibility and efficacy of this intervention. Previous pre‐operative CRC studies have tended to favor multimodal prehabilitation, including aspects of resistance and endurance training, dietary supplementation, and psychological support, but this approach has not been effective in improving pre‐operative fitness, nor improving clinical outcomes.^2^, ^9^, ^11^ In addition, these studies have trained patients for up to 6 weeks,^3^, ^9^, ^10^, ^11^, ^12^ making any observed improvements difficult to translate into clinical practice in the UK due to the aforementioned target time frame.^8^ Many other countries are free of the UK NHS time constraints for treatment of cancer; however, management of malignant disease in all countries must be expedited to ensure best outcomes for patients. Therefore, pre‐operative exercise programs must be effective in a short time frame.

High‐intensity interval training (HIIT) is generally characterized as brief bouts of intense effort interspersed with rest or active recovery.^13^ To date, the term “HIIT” has been applied to a wide range of exercise protocols with varying rest and “recovery” profiles, but is most commonly used to describe brief (45 seconds to 2 minutes) episodes of high but not maximal intensity exercise interspersed with rest or activity recovery. This can be compared to sprint interval training (SIT) where maximal, “all‐out” intervals feature. These types of interval‐based training regimes have long been used to train athletic populations^14^ and have more recently been applied to clinical populations in an attempt to improve various aspects of health.^15^, ^16^ In clinical populations, reduced‐intensity HIIT protocols are commonly used as a substitute for the extremely demanding traditional Wingate (SIT‐type) protocols^17^; these modified protocols continue to show significant improvements in physiological parameters associated with health, despite lower exercise loads.^18^

When considering NHS cancer waiting‐time targets, a distinct benefit of HIIT is the rapidity of improvements in fitness compared to endurance exercise training.^19^ For example, in just 28 days, 5 × 1‐minute HIIT has been shown to improve VO_2_ peak by an average of 2.3 mL/kg/min in a healthy volunteer group age‐matched to colorectal cancer patients.^20^ A 1.5 mL/kg/min increase in AT has been reported as clinically relevant in patients waiting for surgery, with improvements of this magnitude shown to move one third of pre‐operative cancer patients from high perioperative risk to a lower risk group.^21^ A further marked benefit of HIIT is the time efficiency (per session) in establishing improvements in fitness. One of the most commonly stated barriers to exercise is “lack of time”,^22^, ^23^ which is likely to be especially true in pre‐operative patients who are trying to attend to many aspects of their social, professional, and personal lives prior to the hiatus imposed by surgery.

In summary, there is a substantial evidence base that (a) patients with higher CRF do better after surgery, (b) HIIT can elicit rapid improvements in CRF, and (c) our specific HIIT protocol has been shown to be effective in a healthy older population age‐matched to those most commonly presenting for CRC surgery. Therefore, the aim of this study was to test the effectiveness of a specific HIIT protocol for improving CRF in pre‐operative CRC patients within the UK 31‐day cancer treatment waiting‐time target.

## 2 METHODS

This was a prospective, cohort, intervention study designed to test the feasibility and effectiveness of our particular HIIT intervention in a specific patient group and associated time frame. Ethical approval for this study was obtained from NRES, East Midlands (14/EM/1131). The study was registered with clinicaltrials.gov (NCT02188342), and all study procedures complied with the 1983 Declaration of Helsinki.

### 2.1 Patient recruitment

Twenty‐four patients were recruited from a single‐center CRC multidisciplinary team meeting (MDT) over a 13‐month period. All patients with CRC who were: (a) recommended surgery without neoadjuvant treatment, (b) able to give informed consent, and (c) between 18 and 98 years of age, were identified as potential study participants and as such had the study introduced to them at their first outpatient clinic following MDT. Patients then received a phone call to determine whether they were interested in study participation, and if so, were invited for a screening visit. This screening visit involved the following: (a) obtaining full written informed consent, (b) documenting past medical history, (c) a cardiorespiratory clinical examination, (d) resting blood pressure measurements, and (e) an electrocardiogram (ECG).

The only exclusion criteria for this study were those for clinical safety as recommended by the American Thoracic Society CPET Guidelines,^24^ and inability to give informed consent.

All study visits (including HIIT sessions) took place at the University of Nottingham Royal Derby Hospital Centre. Inclusion in the study did not affect the patients’ clinical pathway in that surgery was not delayed to accommodate the study and all clinical management was in line with normal standards of care.

### 2.2 Study visits

The first study visit (after screening) was a baseline assessment session which consisted of demographic data collection, cardiovascular measurements (resting blood pressure and heart rate), and a CPET. The CPET protocol was identical to that previously used by our research group.^20^ In brief, CPET was performed on a Lode Corival cycle ergometer (Lode Corival, Lode) with in‐line gas analysis system (ZAN 680, nSpire Health), using a standard 15‐30 W per minute ramp protocol based on participants’ (self‐reported) pre‐test fitness, gender, age, and height. Following a 2‐minute period of unloaded cycling, participants were instructed to maintain a cadence of 50‐60 revolutions per minute (rpm) and were verbally encouraged to exercise to >85% of age‐predicted maximal heart rate and to a respiratory exchange ratio (VCO_2_/VO_2_) above 1.0. The test was deemed complete when the participant indicated that they had reached volitional exhaustion. During all CPET assessments, participants were monitored with a 12‐lead ECG, non‐invasive blood pressure monitoring, and pulse oximetry. All sessions were supervised by an advanced life support‐trained clinician with termination criteria taken from the American Thoracic Society/American College of Chest Physicians Statement on CPET.^24^

Forty‐eight to seventy‐two hours prior to surgery (and ~48 hours after the final HIIT session), all patients completed a post‐HIIT assessment visit where all baseline measurements were repeated.

### 2.3 High‐intensity interval training

Following the baseline assessment session, patients attended for a minimum of six HIIT sessions, between 3 and 4 times each week, before surgery. HIIT sessions were performed on a stationary cycle ergometer (Lode Corival, Lode) with 5 × 1‐minute high‐intensity intervals interspersed with 90‐second (unloaded) active recovery. The intensity of the intervals was set between 100% and 120% of the maximum wattage achieved during baseline CPET, determined by a HIIT assessment session. Details of this assessment session are as previously published.^25^ Each HIIT session was preceded by a 2‐minute unloaded warm‐up and finished with a 3.5‐minute unloaded monitoring period.

### 2.4 Statistical analysis

An a priori power calculation, based on our previous research,^20^ suggested that 18 patients would be needed to detect a mean clinically significant change in VO_2_ peak of 2 mL/kg/min^21^ with 80% power and significance at the 5% level. We therefore aimed to recruit 22 individuals in order to achieve 18 complete data sets based on an assumed dropout rate of 20% (in keeping with our previous studies).

The D'Agostino and Pearson omnibus test was used to confirm normal distribution of the data. Normal data are expressed as mean ± standard deviation (SD), and nonparametric data as median ± interquartile range (IQR). Paired Student's *t* tests (two‐tailed) were used to test parametric data, and the Wilcoxon matched‐pairs signed‐rank test was used for nonparametric data. Pearson's correlation analysis was used to explore the relationship between changes in VO_2_ peak and number of HIIT sessions or baseline fitness. GraphPad Prism 7 was used for data analysis with level of significance set at *P* < 0.05.

## 3 RESULTS

### 3.1 Patient characteristics

**Figure 1:**
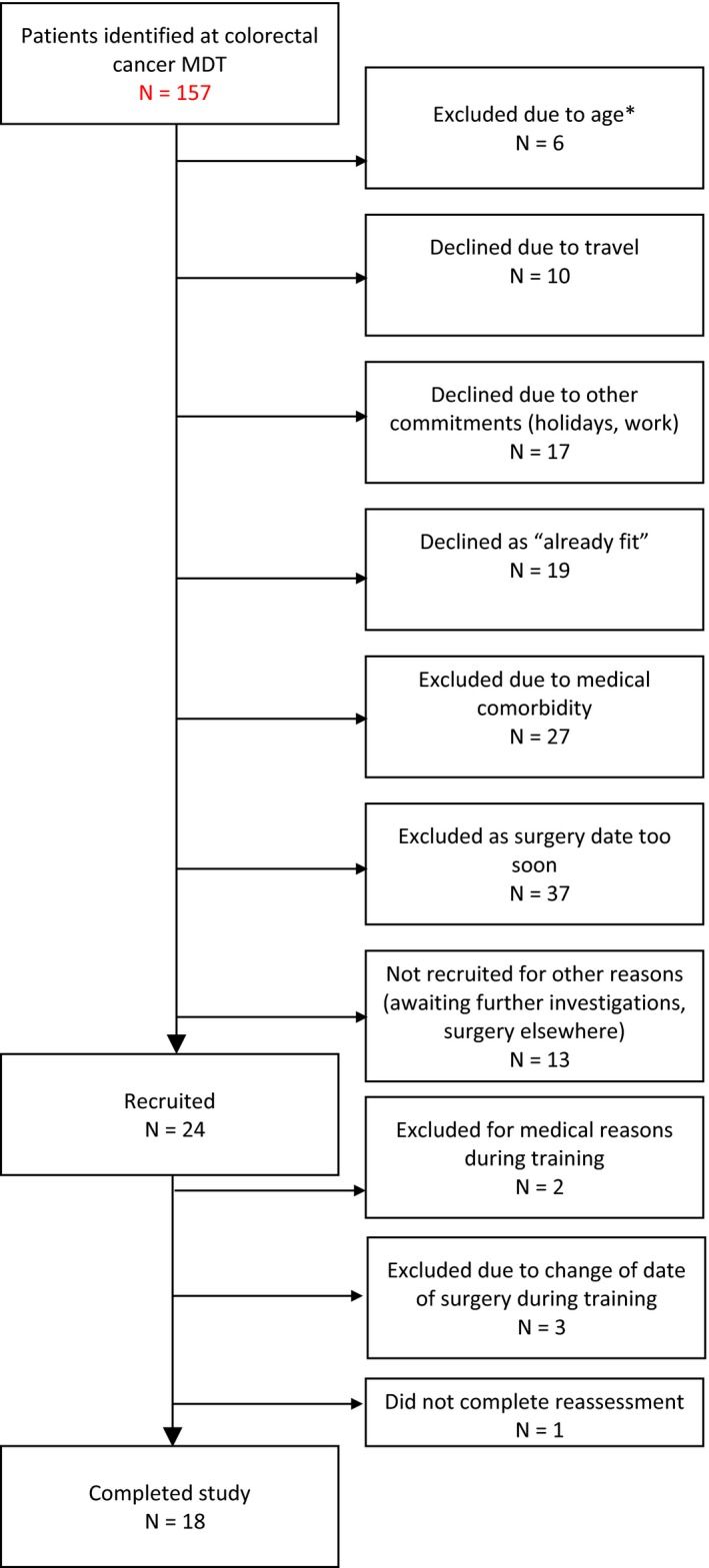
Flow diagram of patients approached to take part in the study. Abbreviations: CPET, cardiopulmonary exercise test; HIIT, high‐intensity interval training; MDT, multidisciplinary team meeting. *Prior to a minor ethics amendment which increased the upper age limit from 75 to 98 y

One hundred and fifty‐seven patients were approached to take part in the study over a 13‐month period (Figure 1). Twenty‐four patients completed the screening process, at which two were excluded from the study due to knee pain which prevented cycling and undiagnosed hypertension, respectively. Three patients were withdrawn after screening as earlier opportunities to operate were found, leaving them without enough time to complete the minimum six HIIT sessions. One patient did not attend for their post‐HIIT reassessment visit. Eighteen patients completed the whole study protocol (Table 1).

**Table 1:** Patient characteristics

Characteristic	Number of participants
Age (SD) (y)	67 (±8)
Gender	13 male: 5 female
Body mass index (BMI) (kg/m^2^)	28.7 (26.6‐31.4)
Habitual exercise sessions per week	<1 per week: 12 2‐3 per week: 3 4‐5 per week: 2 >5 per week: 1
Comorbidities	Myocardial infarction: 2 Prior cancer diagnosis (not current colorectal cancer): 2 Type 2 diabetes: 4 Chronic obstructive pulmonary disease: 1 Anxiety/depression: 2 Controlled hypertension: 2 Musculo‐skeletal history (eg, joint replacement and spinal surgery): 8
Medication	Statin: 5 Isosorbide mononitrate: 1 Inhaled bronchodilator: 2 Warfarin: 1 Metformin: 1 Antihypertensives: 12
Site of cancer	Right colon: 4 Left colon: 5 Rectum: 9
American Society of Anesthesiologists physical status classification grade (excluding cancer)	Grade 1 (normal, healthy): 12 Grade 2 (mild systemic disease): 6
Cancer tumor (T) stage	T1 or polyp cancer: 3 T2: 7 T3: 5 T4: 3
Cancer lymph node (N) stage	N0: 10 N1: 7 N2: 1

### 3.2 High‐intensity interval training

Patients completed a median of 8 (6‐14) HIIT sessions over a mean of 19 (±7) days. The mean training workload was 155 W (±55 W), with all patients training between 100% and 120% of their maximum wattage achieved during baseline CPET. Importantly for a feasibility study, there were no clinically significant adverse events related to HIIT and there was 100% compliance to the HIIT program.

### 3.3 Cardiorespiratory fitness (CRF)

**Figure 2:**
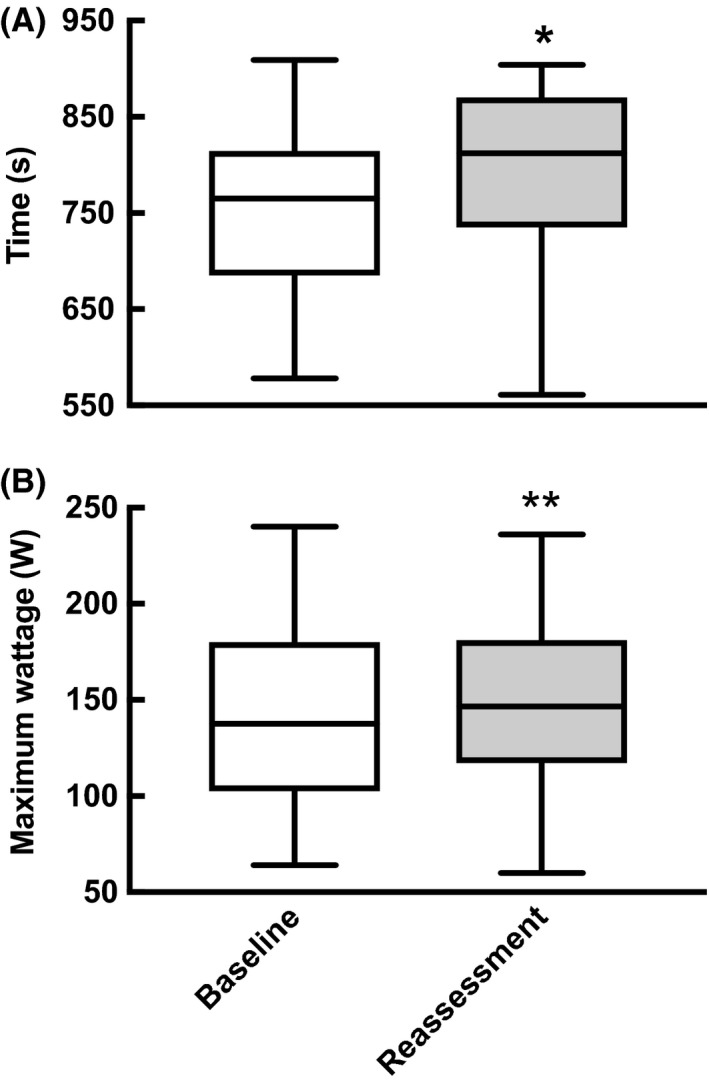
Individual changes in peak oxygen consumption (VO_2_ peak) in colorectal cancer patients (n = 18) after high‐intensity interval training

There was no significant change in absolute VO_2 _peak, VO_2 _peak relative to body weight, or AT (absolute or relative) after HIIT (Table 2). A numerical improvement in VO_2_ peak was seen in 9 of 18 patients, with 6 of these patients improving by >2 mL/kg/min or more, a previously stated value for a clinically relevant improvement,^21^ after HIIT (Figure 2).

**Table 2:** Cardiorespiratory fitness parameters (peak oxygen consumption (VO_2_ peak) and anaerobic threshold (AT)) as determined by cardiopulmonary exercise testing (CPET) in pre‐operative colorectal cancer patients (N = 18) before (baseline) and after (reassessment) high‐intensity interval training

	Baseline CPET	Reassessment CPET	*P*‐value
VO_2_ peak (L/min)	2.1 ± 0.6	2.1 ± 0.6	0.94
VO_2 _peak (mL/kg/min)	23.9 ± 7.0	24.2 ± 7.8	0.47
AT (L/min)	1.2 ± 0.3	1.2 ± 0.3	0.80
AT (mL/kg/min)	14.0 ± 3.4	14.5 ± 4.5	0.50

Note

Data are presented as absolute (L/min) and relative (to body weight) values (mL/kg/min). Analysis was performed by paired Student's *t* test.

Although there was variation in the number of HIIT sessions completed by each patient due to factors out with our control (eg, revision of operation date and available time compressed to <31 days to meet the 62‐day general practitioner referral to first treatment cancer target^26^), there was no significant relationship between the number of HIIT sessions completed and the magnitude of change in CRF (absolute VO_2_ peak (L/min): *r* ^2 ^= 0.007, *P* = 0.74; absolute AT (L/min): *r* ^2 ^= 0.116, *P* = 0.17; relative (to body weight) VO_2_ peak (mL/kg/min): *r* ^2 ^= 0.007, *P* = 0.74; and relative AT (mL/kg/min): *r* ^2 ^= 0.116, *P* = 0.17). Similarly, there was no significant relationship between baseline fitness and change in CRF, with this true for both absolute (VO_2_ peak (mL/min): *r* ^2 ^= 0.022, *P* = 0.56; AT (mL/min): *r* ^2 ^= 0.13, *P* = 0.14) and relative (VO_2_ peak (mL/kg/min): *r* ^2 ^= 0.020, *P* = 0.58; AT (mL/kg/min): *r* ^2 ^= 0.13, *P* = 0.14) values.

In addition to values obtained at volitional exhaustion (ie, the end of the CPET), submaximal heart rate and O_2_ pulse were also assessed at 25% and 50% wattage of the baseline CPET. Heart rate was significantly lower at all intensities during the reassessment CPET, but this was not reflected in the O_2_ pulse measurements where no significant changes were observed after HIIT (Table 3).

**Table 3:** Maximal and submaximal heart rate (HR) and O_2_ pulse values at set percentages of maximum wattage from the baseline cardiopulmonary exercise test (CPET) in pre‐operative colorectal cancer patients (N = 18) before (baseline) and after (reassessment) high‐intensity interval training

	Baseline CPET	Reassessment CPET	*P*‐value
HR at 25% (bpm)	90 (±19)	86 (±17)	0.008
HR at 50% (bpm)	103 (±19)	98 (±17)	0.003
HR at 100% (bpm)	138 (±22)	130 (±27)	0.01
O_2_ pulse at 25%	9.9 (±2.3)	9.3 (±2.5)	0.25
O_2_ pulse at 50%	12.9 (±3.0)	11.9 (±3.13)	0.05
O_2_ pulse at 100%	14.8 (±2.86)	14.9 (±3.27)	0.86

Note

Analysis was performed by paired Student's *t* test.

### 3.4 Exercise performance

**Figure 3:**
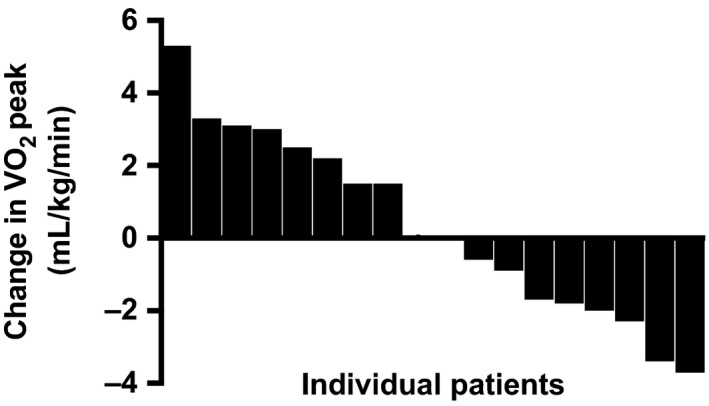
Cardiopulmonary exercise testing (CPET) time to failure and wattage at failure before (baseline) and after (reassessment) high‐intensity interval training in pre‐operative colorectal cancer patients (N = 18). Analysis was performed by paired Student's *t* test; *P < 0.05, **P < 0.01

Despite a lack of improvement in CRF parameters after HIIT, there was a significant increase in CPET time to failure (752 ± 93 vs 789 ± 99 seconds, *P* = 0.02) and correspondingly CPET maximum wattage (142 ± 50 W vs 150 ± 49 W, *P* = 0.007) (Figure 3).

### 3.5 Cardiovascular parameters

Resting systolic blood pressure was significantly reduced after HIIT (Table 4), although no changes in diastolic blood pressure or mean arterial pressure were observed.

**Table 4:** Resting cardiovascular parameters (systolic blood pressure (SBP), diastolic blood pressure (DBP), and mean arterial pressure (MAP) and resting heart rate (RHR)) in pre‐operative colorectal cancer patients (N = 18) before (baseline) and after (reassessment) high‐intensity interval training

	Baseline	Reassessment	*P*‐value
SBP (mm Hg)	152 (±19)	142 (±19)	0.0005
DBP (mm Hg)	81 (±10)	81 (±14)	0.05
MAP (mm Hg)	105 (±10)	101 (±14)	0.19
RHR (bpm)	75 (±16)	75 (±14)	0.87

Note

Analysis was performed by paired Student's *t* test.

## 4 DISCUSSION

This study demonstrates that it is not feasible to increase the CRF of pre‐operative CRC patients in a pragmatic, real‐world setting within UK NHS cancer waiting times using our specific 5 × 1‐minute HIIT protocol.

Despite not being able to improve CRF, our HIIT protocol did lead to a significant reduction in resting systolic blood pressure, an important physiological parameter which is independently associated with reduced overall mortality, coronary events, and stroke in older individuals.^27^ In addition, our CRC patients displayed improved exercise efficiency (a lower heart rate for matched power outputs) and exercise performance (maximum wattage and time to failure during CPET) after HIIT. This improvement in energy efficiency in response to a physiological demand may prove important when patients need to meet the increased energy demands of the perioperative phase.^28^ It must also be acknowledged that there may be other benefits of this pre‐operative exercise that were not measured during this study such as improved health‐related quality of life. This has been shown with many exercise strategies in cancer patients during active treatment.^29^

Although the underlying mechanisms affecting the magnitude of gains in CRF in these patients are not known, propositions based on previous findings can be made. Firstly, previous work in CRC patients has shown that AT significantly improves after resection of the cancer without a change in lean muscle mass or self‐reported physical activity.^30^ This suggests that physiological factors associated with the presence of a cancer (such as inflammation^31^ and impaired mitochondrial function^32^) may lead to a blunting of the expected adaptive responses to exercise training while the cancer remains in situ. This notion is supported by additional work in both rodent and human studies. In pre‐clinical, rodent work, it has been shown that the increased inflammatory load associated with cancer significantly decreases muscle oxidative capacity and mitochondrial dynamics,^33^ potentially decreasing adaptive responses to exercise.^18^ In humans, rates of muscle protein synthesis (MPS) in response to the anabolic stimulus of feeding are blunted in CRC patients while the cancer is in situ, but restored upon resection.^32^ Given that feeding and muscle contraction are well established as the two most potent anabolic stimuli for MPS^34^ and that an exercise × nutrition interaction is required for exercise to act as a positive anabolic stimulus to achieve muscle hypertrophy, the known anabolic blunting to nutrition in the myofibrillar muscle fraction caused by a tumor burden may well extend to anabolic blunting to exercise in both myofibrillar and mitochondrial subfractions, supporting the notion that a cancer in situ has the potential to cause disruption of adaptive metabolic pathways.

An additional concept to consider is that of exercise responder status. It is emerging from the literature that large interindividual heterogeneity in adaptation exists for almost all forms of exercise training, whereby some people respond much less well to exercise interventions than the mean.^35^, ^36^ Indeed, for a range of exercise training modalities and different primary endpoints (eg, VO_2_ max or insulin sensitivity for aerobic exercise training and muscle hypertrophy for resistance exercise training) it has been shown that ~20% do not show numerical improvement.^37^ This phenomenon has also been observed for HIIT, with a similar rate of “non‐responders” for gains in CRF.^25^ However, in this study 50% of individuals demonstrated no numerical improvement in indices of CRF, almost double that observed in healthy volunteer studies.^25^ However, perhaps a better comparison lies with other exercise training studies in pre‐operative cancer patients who report a similar non‐response rate of 50%,^38^ perhaps suggestive of an intrinsic physiological mechanism limiting adaptive potential in these individuals while the cancer is in situ.

Another proposition is related to the known link between physical activity and cancer. Given that physical *in*activity is an independent risk factor for many cancers^39^, ^40^ and it is likely that those who experience positive exercise training gains are more likely to adhere to exercise, our (and others) CRC patient group may have a higher proportion of exercise “non‐responders” than a healthy volunteer cohort, as their inability to make exercise training gains discouraged lifelong exercise and therefore increased their cancer risk.

Our negative finding may be indicative that there is just not enough time in a real‐world setting, constrained by government‐based waiting‐time targets, for our specific exercise protocol to elicit improvements in CRF in this situation. In this study, a mean of eight HIIT sessions was completed in an average of 19 days. Due to screening, baseline, and reassessment visits, the 31‐day NHS cancer waiting‐time target actually allowed only 23 days for HIIT. In a recent systematic review,^41^ only one study applied an (non‐HIIT) exercise intervention for pre‐operative CRC patients within this time frame,^11^ and while they saw improvements in respiratory muscle endurance, in agreement with our findings they did not see evidence of improvements in CRF. Although previous HIIT studies using our exact protocol in healthy older volunteers did report improvements in CRF within a 31‐day window,^20^ all of the healthy volunteers were able to complete a total of 12 HIIT sessions at a frequency of three sessions each week and as such the possibility of being able to improve the CRF of our patients, had we been able to train each patient for the full 31‐days, must be considered. However, our aim was to test the feasibility and effectiveness of this HIIT protocol in a real‐world, target‐constrained clinical environment, and this meant that the time available for pre‐operative exercise was curtailed. Outside the UK, these time constraints are not globally applied and it would therefore be interesting to see whether improvements in CRF in this patient group are possible over longer time frames using this HIIT protocol.

It may also be that our particular HIIT protocol was not optimal to improve CRF in the short time frame that was available to us. Other HIIT studies have shown improvements in CRF in short time frames, albeit in younger individuals^42^ and using protocols that, to our knowledge, have not been assessed for feasibility or efficacy in older adults or specific disease cohorts. Further work should be undertaken to explore exercise modality, training intensity, interval length, and session frequency to try and determine an optimal HIIT protocol to improve the CRF of pre‐operative patients in the short time frame available.

Debate is ongoing as to whether cancer treatment pathways should be altered to allow prehabilitation regimes to be more effective.^43^ Possibilities for this include introducing prehabilitation earlier in the patient journey (eg, at time of referral for investigation of symptoms) or delaying surgery to allow longer for prehabilitation. This first option would have significant resource implications in that large numbers of patients would begin exercise training, the majority of whom would have normal results of their investigations and as such no longer need prehabilitation. The counterargument to the second option is that delays will adversely affect cancer outcomes and surgery should occur at the earliest possible opportunity.^43^ This is despite 31‐day targets being arbitrary, with no evidence to suggest that delaying surgery by a short time leads to worse oncological outcomes.

In summary, HIIT remains a promising intervention for producing rapid improvements in fitness in numerous clinical cohorts,^15^, ^16^, ^44^ but our particular study protocol did not demonstrate an improvement in CRF in CRC patients within the current surgery target times in the UK.

A major strength of this study is its real‐world setting and pragmatism. Research studies are often criticized for their lack of generalizability,^45^ but this study was performed entirely in line with the clinical time frames and treatment pathways in the NHS with limited exclusion criteria. In addition, although this study included a relatively small number of patients, the study was adequately powered using data from studies in a group age‐matched to CRC patients.

A weakness of this study is the cohort design with no control arm. It is possible that although we did not show an improvement in CRF after HIIT, we may have attenuated declines that would have occurred during this period without HIIT. However, declines in fitness have not been seen in the control groups of other prehabilitation studies.^10^, ^21^ Furthermore, the ethics of non‐exercise control groups has been questioned when there is a wealth of supportive evidence in favor of physical activity and improved perioperative outcomes.^46^ In addition, this study was designed to test the feasibility and effectiveness of our HIIT intervention in a specific patient group, endpoints which were wholly achievable without a control group.

## 5 PERSPECTIVE

Our 5 × 1‐minute HIIT prehabilitation regime is not effective for improving CRF before CRC surgery within the real‐world confines of UK cancer surgery target times. Given the time efficiency of HIIT sessions and the rapidity of HIIT‐induced adaptations in other cohorts out with CRC, it seems unlikely that alternative exercise modalities will prove more effective within these constraints, but other HIIT protocols warrant further investigation.

The strong evidence of improved post‐operative outcomes with improved pre‐operative CRF raises the question of whether cancer target times (for treatment) should accommodate pre‐operative exercise programs to potentially facilitate improvements in CRF before surgery. Additionally, public health efforts to improve the fitness of those individuals most likely to develop cancer need to be developed. Of note, a number of individual patients do demonstrate significant improvements in CRF in the limited time available in the UK; with future research, it may become possible to identify those who are likely to respond to surgical exercise prehabilitation.

## References

[1] West MA , Parry MG , Lythgoe D , et al. Cardiopulmonary exercise testing for the prediction of morbidity risk after rectal cancer surgery. Br J Surg. 2014;101(9):1166‐1172. 24916313 10.1002/bjs.9551

[2] Li C , Carli F , Lee L , et al. Impact of a trimodal prehabilitation program on functional recovery after colorectal cancer surgery: a pilot study. Surg Endosc. 2013;27(4):1072‐1082. 23052535 10.1007/s00464-012-2560-5

[3] Mayo NE , Feldman L , Scott S , et al. Impact of preoperative change in physical function on postoperative recovery: argument supporting prehabilitation for colorectal surgery. Surgery. 2011;150(3):505‐514. 21878237 10.1016/j.surg.2011.07.045

[4] Moran J , Wilson F , Guinan E , McCormick P , Hussey J , Moriarty J . Role of cardiopulmonary exercise testing as a risk‐assessment method in patients undergoing intra‐abdominal surgery: a systematic review. Br J Anaesth. 2016;116(2):177‐191. 26787788 10.1093/bja/aev454

[5] West MA , Lythgoe D , Barben CP , et al. Cardiopulmonary exercise variables are associated with postoperative morbidity after major colonic surgery: a prospective blinded observational study. Br J Anaesth. 2014;112(4):665‐671. 24322573 10.1093/bja/aet408

[6] Wasserman K . The anaerobic threshold: definition, physiological significance and identification. Adv Cardiol. 1986;35:1‐23. 3551513

[7] Ross R , Blair SN , Arena R , et al. Importance of assessing cardiorespiratory fitness in clinical practice: a case for fitness as a clinical vital sign: a scientific statement from the American Heart Association. Circulation. 2016;134:1‐48. 10.1161/CIR.0000000000000461 27881567

[8] Department of Health . The NHS Cancer Plan. London, UK: Department of Health; 2000:1‐98.

[9] Gillis C , Li C , Lee L , et al. Prehabilitation vs rehabilitation: a randomized control trial in patients undergoing colorectal resection for cancer. Anaesthesiology. 2014;121(5):937‐947. 10.1097/ALN.0000000000000393 25076007

[10] Kim DJ , Mayo NE , Carli F , Montgomery DL , Zavorsky GS . Responsive measures to prehabilitation in patients undergoing bowel resection surgery. Tohoku J Exp Med. 2009;217(2):109‐115. 19212103 10.1620/tjem.217.109

[11] Dronkers JJ , Lamberts H , Reutelingsperger I , et al. Preoperative therapeutic programme for elderly patients scheduled for elective abdominal oncological surgery: a randomized controlled pilot study. Clin Rehabil. 2010;24(7):614‐622. 20530651 10.1177/0269215509358941

[12] Carli F , Charlebois P , Stein B , et al. Randomized clinical trial of prehabilitation in colorectal surgery. Br J Surg. 2010;97(8):1187‐1197. 20602503 10.1002/bjs.7102

[13] Gibala M , McGee SL . Metabolic adaptations to short‐term high‐intensity interval training: a little pain for a lot of gain? Exerc Sport Sci Rev. 2008;36(2):58‐63. 18362686 10.1097/JES.0b013e318168ec1f

[14] Laursen PB , Jenkins DG . The scientific basis for high‐intensity interval training: optimising training programmes and maximising performance in highly trained endurance athletes. Sports Med. 2002;32(1):53‐73. 11772161 10.2165/00007256-200232010-00003

[15] Ciolac E . Review article: high‐intensity interval training and hypertension maximising the benefits of exercise? Am J Cardiovasc Dis. 2012;2(2):102‐110. 22720199 PMC3371620

[16] Liou K , Ho S , Fildes J , Ooi S‐Y . High intensity interval vs moderate intensity continuous training in patients with coronary artery disease: a meta‐analysis of physiological and clinical parameters. Hear Lung Circ. 2016;25(2):166‐174. 10.1016/j.hlc.2015.06.828 26375499

[17] Little JP , Safdar A , Wilkin GP , Tarnopolsky MA , Gibala MJ . A practical model of low‐volume high‐intensity interval training induces mitochondrial biogenesis in human skeletal muscle: potential mechanisms. J Physiol. 2010;588(Pt 6):1011‐1022. 20100740 10.1113/jphysiol.2009.181743 PMC2849965

[18] Gibala MJ , Little JP , Macdonald MJ , Hawley JA Physiological adaptations to low‐volume, high‐intensity interval training in health and disease. J Physiol. 2012;590(Pt 5):1077‐1084. 22289907 10.1113/jphysiol.2011.224725 PMC3381816

[19] Milanović Z , Sporiš G , Weston M . Effectiveness of high‐intensity interval training (HIT) and continuous endurance training for VO_2 _max improvements: a systematic review and meta‐analysis of controlled trials. Sport Med. 2015;45:1469‐1481. 10.1007/s40279-015-0365-0 26243014

[20] Boereboom CL , Phillips BE , Williams JP , Lund JN . A 31‐day time to surgery compliant exercise training programme improves aerobic health in the elderly. Tech Coloproctol. 2016;20(6):375‐382. 27015678 10.1007/s10151-016-1455-1

[21] Dunne D , Jack S , Jones RP , et al. Randomized clinical trial of prehabilitation before planned liver resection. Br J Surg. 2016;103(5):504‐512. 26864728 10.1002/bjs.10096

[22] Stutts WC . Physical activity determinants in adults. Perceived benefits, barriers, and self efficacy. AAOHN J. 2002;50(11):499‐507. 12465206

[23] Trost S , Owen N , Bauman A , Sallis J , Brown W . Correlates of adult's participation in physical activity: review and update. Med Sci Sports Exerc. 2002;34(12):1996‐2001. 12471307 10.1097/00005768-200212000-00020

[24] Weisman IM , Marciniuk D , Martinez FJ , et al. ATS/ACCP statement on cardiopulmonary exercise testing. Am J Respir Crit Care Med. 2003;167(2):211‐277. 12524257 10.1164/rccm.167.2.211

[25] Phillips B , Kelly B , Lilja M , et al. A practical and time‐efficient high‐intensity interval training programme modifies cardio‐metabolic risk‐factors in adults at risk for developing type II diabetes. Front Endocrinol (Lausanne). 2017;8:229. 28943861 10.3389/fendo.2017.00229 PMC5596071

[26] National Cancer Action Team , National Cancer Intelligence Network . Cancer Waiting Times: A Guide (version 7); 2007.

[27] Staessen JA , Gasowski J , Wang JG , et al. Risks of untreated and treated isolated systolic hypertension in the elderly: meta‐analysis of outcome trials. Lancet. 2000;355(9207):865‐872. 10752701 10.1016/s0140-6736(99)07330-4

[28] Desborough JP . The stress response to trauma and surgery. Br J Anaesth. 2000;85(1):109‐117. 10927999 10.1093/bja/85.1.109

[29] Mishra SI , Scherer RW , Snyder C , Geigle PM , Berlanstein DR , Topaloglu O . Exercise interventions on health‐related quality of life for people with cancer during active treatment. Cochrane Database Syst Rev. 2012;15(8):CD008465. 10.1002/14651858.CD008465.pub2 PMC7389071 22895974

[30] Williams JP , Nyasavajjala SM , Phillips BE , Chakrabarty M , Lund JN . Surgical resection of primary tumour improves aerobic performance in colorectal cancer. Eur J Surg Oncol. 2014;40(2):220‐226. 24332580 10.1016/j.ejso.2013.11.009

[31] Coussens LM , Werb Z . Inflammation and cancer. Nature. 2002;420:860. 12490959 10.1038/nature01322 PMC2803035

[32] Phillips BE , Smith K , Liptrot S , et al. Effect of colon cancer and surgical resection on skeletal muscle mitochondrial enzyme activity in colon cancer patients: a pilot study. J Cachexia Sarcopenia Muscle. 2013;4(1):71‐77. 22648738 10.1007/s13539-012-0073-7 PMC3581615

[33] White JP , Baltgalvis KA , Puppa MJ , Sato S , Baynes JW , Carson JA . Muscle oxidative capacity during IL‐6‐dependent cancer cachexia. Am J Physiol Regul Integr Comp Physiol. 2011;300(2):R201‐R211. 21148472 10.1152/ajpregu.00300.2010 PMC3043802

[34] Atherton PJ , Smith K . Muscle protein synthesis in response to nutrition and exercise. J Physiol. 2012;590(5):1049‐1057. 22289911 10.1113/jphysiol.2011.225003 PMC3381813

[35] Bouchard C , Antunes‐Correa LM , Ashley EA , et al. Personalized preventive medicine: genetics and the response to regular exercise in preventive interventions. Prog Cardiovasc Dis. 2015;57(4):337‐346. 25559061 10.1016/j.pcad.2014.08.005 PMC4285566

[36] Phillips BE , Williams JP , Gustafsson T , et al. Networks of human muscle adaptation to exercise and age. PLoS Genet. 2013;9(3):e1003389. 23555298 10.1371/journal.pgen.1003389 PMC3605101

[37] Gurd BJ , Giles MD , Bonafiglia JT , et al. Incidence of nonresponse and individual patterns of response following sprint interval training. Appl Physiol Nutr Metab. 2016;41(3):229‐234. 26854820 10.1139/apnm-2015-0449

[38] Huang GH , Ismail H , Murnane A , Kim P , Riedel B . Structured exercise program prior to major cancer surgery improves cardiopulmonary fitness: a retrospective cohort study. Support Care Cancer. 2016;24(5):2277‐2285. 26590843 10.1007/s00520-015-3028-7

[39] Harriss D , Cable N , George K , Reilly T , Renehan A , Haboubi N . Physical activity before and after diagnosis of colorectal cancer: disease risk, clinical outcomes, response pathways and biomarkers. Sport Med. 2007;37(11):947‐960. 10.2165/00007256-200737110-00003 17953466

[40] Warburton D , Nicol CW , Bredin S . Health benefits of physical activity: the evidence. Can Med Assoc J. 2006;174(6):801‐809. 16534088 10.1503/cmaj.051351 PMC1402378

[41] Boereboom C , Doleman B , Lund J , Williams J . Systematic review of pre‐operative exercise in colorectal cancer patients. Tech Coloproctol. 2016;20(2):81‐89. 26614304 10.1007/s10151-015-1407-1

[42] Raleigh J , Giles M , Scribbans T , et al. The impact of work‐matched interval training on V̇O2peak and V̇O2 kinetics: diminishing returns with increasing intensity. Appl Physiol Nutr Metab. 2016;41(7):706‐713. 27337599 10.1139/apnm-2015-0614

[43] Leong K , Chapman M . Current data about the benefit of prehabilitation for colorectal cancer patients undergoing surgery are not sufficient to alter the NHS cancer waiting targets. Color Dis. 2017;19(6):522‐524. 10.1111/codi.13723 28498541

[44] Gillen JB , Little JP , Punthakee Z , Tarnopolsky MA , Riddell MC , Gibala MJ . Acute high intensity interval exercise reduces the postprandial glucose response and prevalence of hyperglycaemia in patients with type 2 diabetes. Diabetes Obes Metab. 2012;14:575‐577. 22268455 10.1111/j.1463-1326.2012.01564.x

[45] Patsopoulos NA . A pragmatic view on pragmatic trials. Dialogues Clin Neurosci. 2011;13(2):217‐224. 21842619 10.31887/DCNS.2011.13.2/npatsopoulos PMC3181997

[46] Dronkers J , Witteman B , van Meeteren N . Surgery and functional mobility: doing the right thing at the right time. Tech Coloproctol. 2016;20(6):339‐341. 27170281 10.1007/s10151-016-1487-6

